# Celastrol Inhibits the Growth of Ovarian Cancer Cells *in vitro* and *in vivo*

**DOI:** 10.3389/fonc.2019.00002

**Published:** 2019-01-28

**Authors:** Li-Na Xu, Na Zhao, Jin-Yan Chen, Piao-Piao Ye, Xing-Wei Nan, Hai-Hong Zhou, Qi-Wei Jiang, Yang Yang, Jia-Rong Huang, Meng-Ling Yuan, Zi-Hao Xing, Meng-Ning Wei, Yao Li, Zhi Shi, Xiao-Jian Yan

**Affiliations:** ^1^Department of Gynecology, The First Affiliated Hospital of Wenzhou Medical University, Wenzhou, China; ^2^Department of Gynecology, The Second Affiliated Hospital of Zhejiang University School of Medicine, Hangzhou, China; ^3^Department of Cell Biology and Institute of Biomedicine, National Engineering Research Center of Genetic Medicine, Guangdong Provincial Key Laboratory of Bioengineering Medicine, College of Life Science and Technology, Jinan University, Guangzhou, China; ^4^Center for Uterine Cancer Diagnosis & Therapy Research of Zhejiang Province, Women's Hospital and Institute of Translational Medicine, Zhejiang University School of Medicine, Zhejiang, China

**Keywords:** celastrol, reactive oxygen species, N-acetyl-cysteine, apoptosis, ovarian cancer

## Abstract

Celastrol is a natural triterpene isolated from the Chinese plant Thunder God Vine with potent antitumor activity. However, the effect of celastrol on the growth of ovarian cancer cells *in vitro* and *in vivo* is still unclear. In this study, we found that celastrol induced cell growth inhibition, cell cycle arrest in G2/M phase and apoptosis with the increased intracellular reactive oxygen species (ROS) accumulation in ovarian cancer cells. Pretreatment with ROS scavenger N-acetyl-cysteine totally blocked the apoptosis induced by celastrol. Additionally, celastrol inhibited the growth of ovarian cancer xenografts in nude mice. Altogether, these findings suggest celastrol is a potential therapeutic agent for treating ovarian cancer.

## Introduction

Ovarian cancer is the most lethal gynecologic cancer and the fifth leading cause of female cancer-related deaths in the United States in 2018 (1). Because of the late stage diagnoses, the prognosis of ovarian cancer remains poor, despite advances in aggressive surgery and combination chemotherapy (2–4). Current treatments for ovarian cancer are far from satisfactory, therefore it is of considerable interest to develop novel therapeutic agents to improve the outcomes of ovarian cancer.

Celastrol is a natural triterpene isolated from the Chinese plant Thunder God Vine (*Tripterygium wilfordii*),which has been reported with a wide range of bioactivities, such as antitumor (5), anti-inflammatory (6), antidiabetic activities (7) and antihypertensive (8). Celastrol has shown the potent antitumor activity in various cancers including prostate, breast, liver, colon, and lung (9–13). Although celastrol is able to induce apoptosis and inhibit proliferation, migration and invasion in ovarian cancer cells *in vitro* (14–16), the effect of celastrol on the growth of ovarian cancer cells *in vivo* is still unknown. Here, we have comprehensively investigated the antitumor activity of celastrol in ovarian cancer cells *in vitro* and *in vivo*.

## Materials and Methods

### Cells Lines and Reagents

The human ovarian cancer lines A2780 and SKOV3 were cultured in Dulbecco's modified Eagle's medium (DMEM) supplemented with 10% fetal bovine serum (FBS), penicillin (100 U/ml) and streptomycin (100 ng/ml) at 37°C with 5% CO2 in a humidified incubator. Celastrol was purchased from Shanghai Tauto Biotechnology. N-acetyl-L-cysteine (NAC) and dihydroethidium (DHE) were purchased from Sigma-Aldrich. Methythiazolyldiphenyl-tetrazolium bromide (MTT), propidium iodide (PI) and other chemicals were purchased from Shanghai Sangon Biotech. Anti-p27 (610241), Anti-Cyclin B1 (554177), and Anti-Cyclin E (51-1459GR) antibodies were from BD Biosciences. Anti-RAF1 (SC-133) antibodies were from Santa Cruz Biotechnology. Anti-PARP (9542), Anti-AKT (4691), Anti-pAKT S473 (4060), Anti-ERK (4695), Anti-pERK T202/T204 (4370), Anti-JNK (9252), Anti-pJNK T183/Y185 (4668), Anti-p38 (9212), Anti-pp38 T180/Y182 (4511) antibodies were from Cell Signaling Technologies. Anti-GAPDH (LK9002T) antibodies were from Tianjin Sungene Biotech.

### MTT Assay

Cells were seeded into a 96-well plate at a density of 0.5 × 10^4^ cells/well. Then, different concentrations of celastrol (10 μL/well) were added to designated wells. After 72 h, 10 μL of MTT was added to each well at a final concentration of 0.5 mg/ml, and the plate was further incubated for 4 h, allowing viable cells to change the yellow MTT into dark-blue formazan crystals. Subsequently, the medium was discarded and 50 μL of dimethylsulfoxide (DMSO) was added to each well to dissolve the formazan crystals. The absorbance in individual well was determined at 570 nm by multidetection microplate reader 680 (BioRad, PA, USA). The concentrations required to inhibit growth by 50% (IC_50_) were calculated from survival curves using the Bliss method (17).

### Cell Cycle Analysis

Cells were harvested and washed twice with cold PBS and then fixed with 70% ice-cold ethanol at 4°C for 30 min. After centrifugation at 200 × g for 10 min, cells were washed twice with PBS, resuspended with 0.5 mL PBS containing PI (50 μg/mL), Triton X-100(0.1%, v/v), 0.1% sodium citrate, and DNase-free RNase (100 μg/mL), and detected by flow cytometry (FCM) after 15 min incubation in the dark at room temperature. Fluorescence was measured at an excitation wave length of 480 nm through a FL-2 filter. Data were analyzed using ModFit LT 3.0 software (Becton Dickinson) (18, 19).

### Apoptosis Analysis

Cell apoptosis was evaluated with FCM assay. Briefly, cells were harvested and washed twice with cold PBS, then stained with Annexin V-FITC and PI in the binding buffer, and detected by FACSCalibur FCM (BD, CA, USA) after 15 min incubation in the dark at room temperature. Fluorescence was measured at an excitation wave length of 480 nm through FL-1 (530 nm) and FL-2 (585 nm) filters. The early apoptotic cells (Annexin V positive only) and late apoptotic cells (Annexin V and PI positive) were quantified (20).

### Western Blot Analysis

Cells were harvested and washed twice with cold PBS and then resuspended and lysed in RIPA buffer (1% NP-40, 0.5% sodium deoxycholate, 0.1% SDS, 10 ng/mL PMSF, 0.03% aprotinin, and 1 μM sodium orthovanadate) at 4°C for 30 min. Lysates were centrifuged at 14,000 × g for 10 min and supernatants were collected. Proteins were separated on 12% SDS-PAGE gels and transferred to polyvinylidene difluoride membranes. Membranes were blocked with 5% BSA and incubated with the indicated primary antibodies. Corresponding horseradish peroxidase-conjugated secondary antibodies were used against each primary antibody. Proteins were detected using the chemiluminescent detection reagents and films (21, 22).

### Reactive Oxygen Species Assay

Cells were incubated with 10 μM of DHE at 37°C for 30 min, washed twice with PBS, and microphotographed under a conventional fluorescent microscope (Olympus, Japan) immediately. For each well, 5 fields were taken randomly. Then, cells were rapidly digested, harvested and washed twice with cold PBS, and detected by FCM. The DHE Fluorescence intensity was measured and quantified at an excitation wave length of 518 nm through PE filters (23, 24).

### Nude Mice Xenograft Assay

Balb/c nude mice were obtained from the Guangdong Medical Laboratory Animal Center and maintained with sterilized food and water. This study was carried out in accordance with the recommendations of the Guidelines for the Care and Use of Laboratory Animals, and the protocol were approved by the Institutional Animal Care and Use Committee of Jinan University. Four female nude mice with 4–5 weeks old and 20–22 g weight were used for each group. Each mouse was injected subcutaneously with A2780 cells (4 × 10^6^ in 100 μl of medium) under the left and right shoulders. Mice were randomized into two groups, when the subcutaneous tumors were approximately 0.3 × 0.3 cm^2^ (two perpendicular diameters) in size, and were injected intraperitoneally with vehicle alone (0.5% methylcellulose) and celastrol (2 mg/kg) every day. The body weights of mice and the two perpendicular diameters (A and B) of tumors were recorded every day. The tumor volume (V) was calculated as:

$$\begin{array}{l} V=\pi /6{\left(1/2\left(A+B\right)\right)}^{3} \end{array}$$

The mice were anaesthetized after experiment, and tumor tissue was excised from the mice and weighted. The rate of inhibition (IR) was calculated according to the formula:

IR = 1-Mean tumor weight of experimental group/Mean tumor weight of control group × 100% (25)

### Statistical Analysis

A student's *t*-test was used to compare individual data points between two groups. A *P*-value of < 0.05 was set as the criterion for statistical significance.

## Results

### Celastrol Inhibited the Growth of Ovarian Cancer Cells *in vitro*

**Figure 1:**
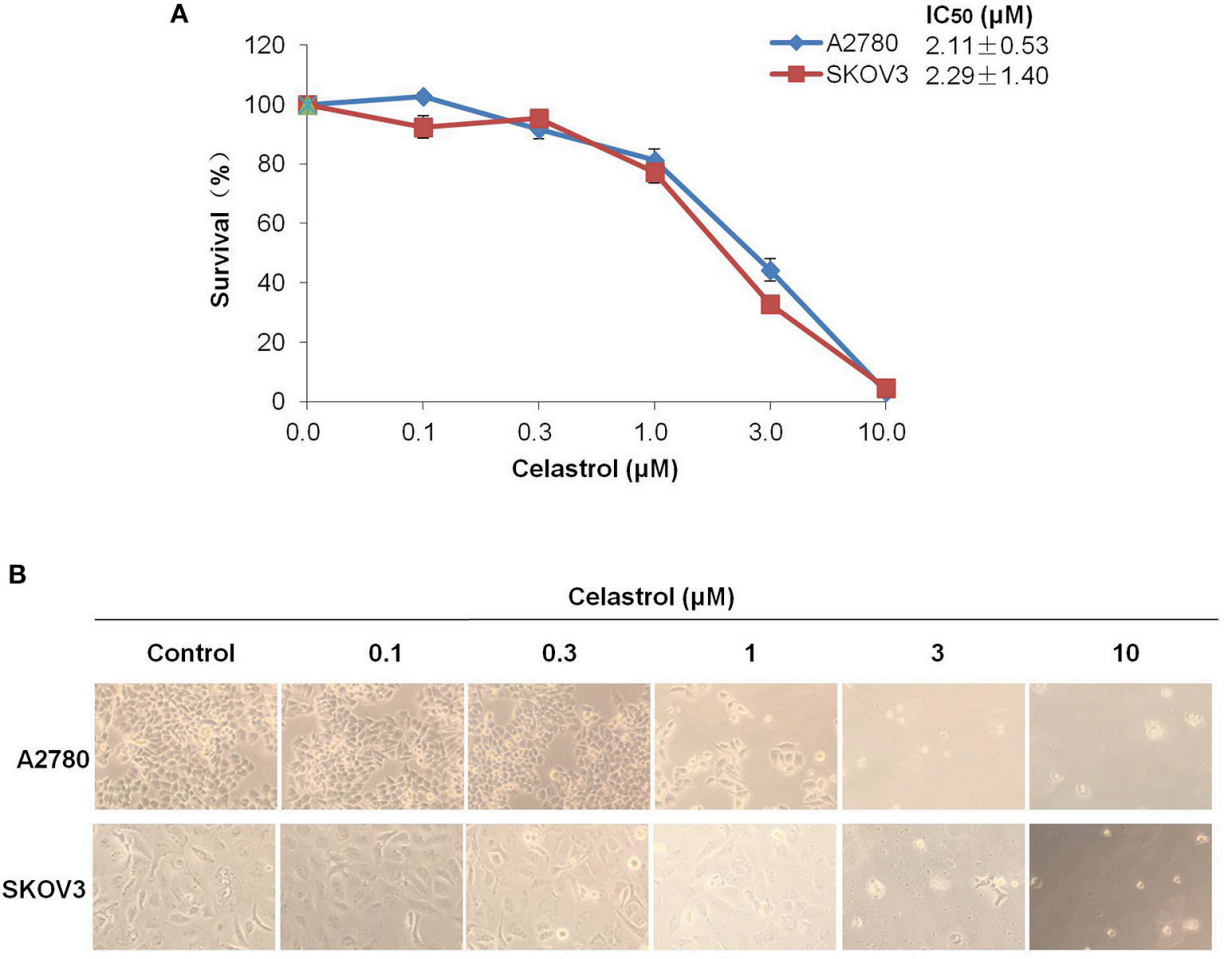
Celastrol inhibited the growth of ovarian cancer cells *in vitro*. **(A)** The growth curves, IC_50_ values and **(B)** phase-contrast images of A2780 and SKOV3 cells treated with the indicated concentrations of celastrol (0, 0.1, 0.3, 1, 3, and 10 μM) for 72 h. Cell survival was measured by MTT assay, and the IC_50_ values of celastrol in each cell lines were calculated.

To access the effect of celastrol on ovarian cancer cells, we treated two ovarian cancer cell lines A2780 and SKOV3 with the increasing concentrations of celastrol range from 0.1 to 10 μM for 72 h. As shown in Figures 1A,B, the results of MTT assay revealed that the growth of two ovarian cancer cell lines was similarly inhibited by celastrol in a dose-dependent manner with the IC_50_ values were 2.11 and 2.29 μM in A2780 and SKOV3 respectively. These data suggested that celastrol inhibits the growth of ovarian cancer cells.

### Celastrol Induced Cell Cycle Arrest in Ovarian Cancer Cells

**Figure 2:**
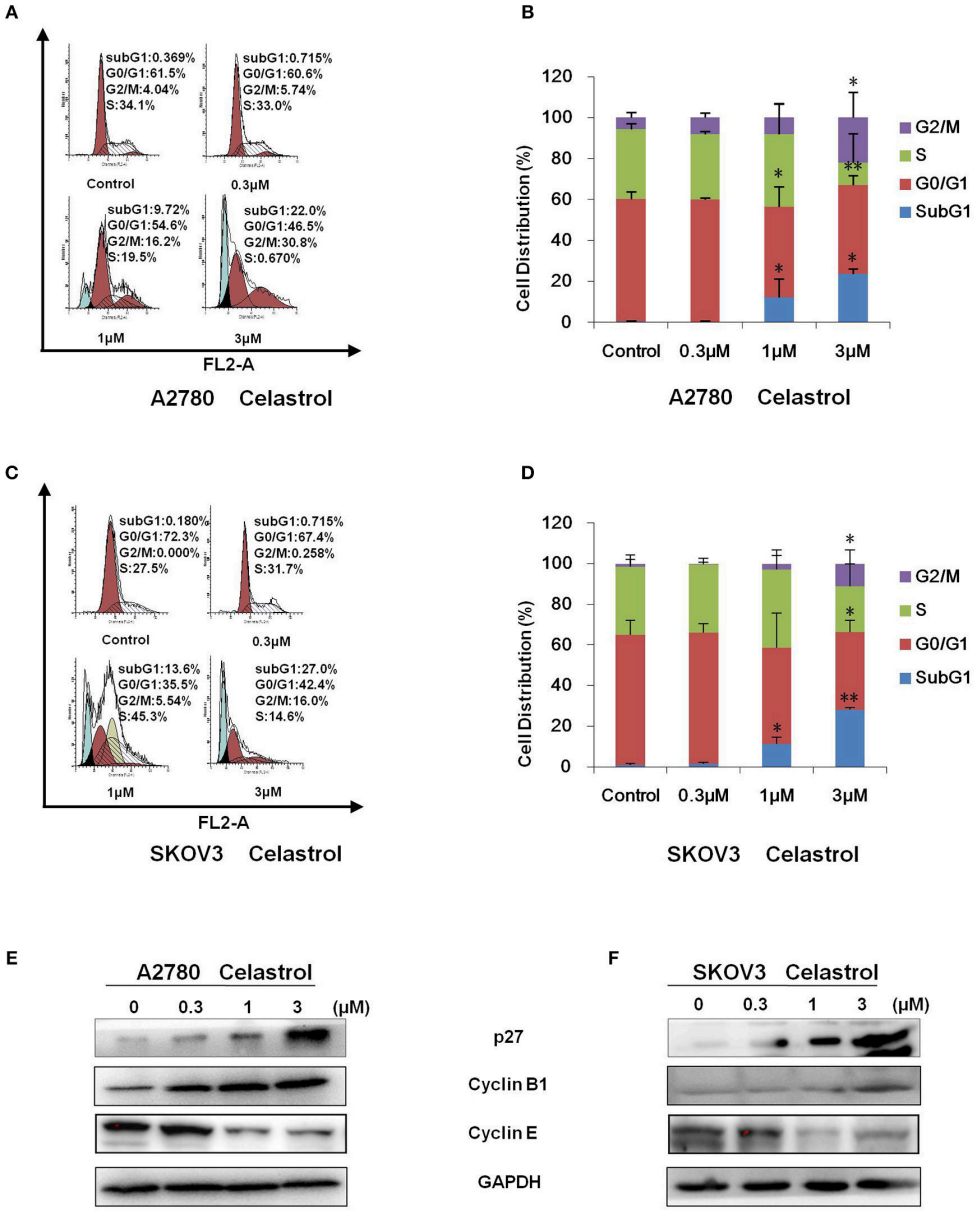
Celastrol induced cell cycle arrest in ovarian cancer cells. A2780 and SKOV3 cells were treated with celastrol with the indicated concentrations for 48 h, then cell cycle was detected by FCM. The representative charts **(A,C)**, quantified data **(B,D)**, and Western blot results **(E,F)** of three independent experiments are shown. ^*^P < 0.05 and ^**^P < 0.01 vs. corresponding control.

To determine whether celastrol is able to induce cell cycle arrest, cell cycle distribution was examined after celastrol treatment. A2780 and SKOV3 cells were treated with 0.3, 1 and 3 μM of celastrol for 48 h, then stained with PI and examined by FCM. As shown in Figures 2A–D, celastrol induced the accumulation in Sub G1 and G2/M phase and reduction in G0/G1 and S phase in two ovarian cancer cell lines. Next, the cell cycle related proteins were detected by Western Blot. As shown in Figures 2E,F, increased p27 and Cyclin B1 and decreased Cyclin E proteins were detected in celastrol-treated A2780 and SKOV3 cells. Together, these results indicated that celastrol induces cell cycle arrest in ovarian cancer cells.

### Celastrol Induced Apoptosis in Ovarian Cancer Cells

**Figure 3:**
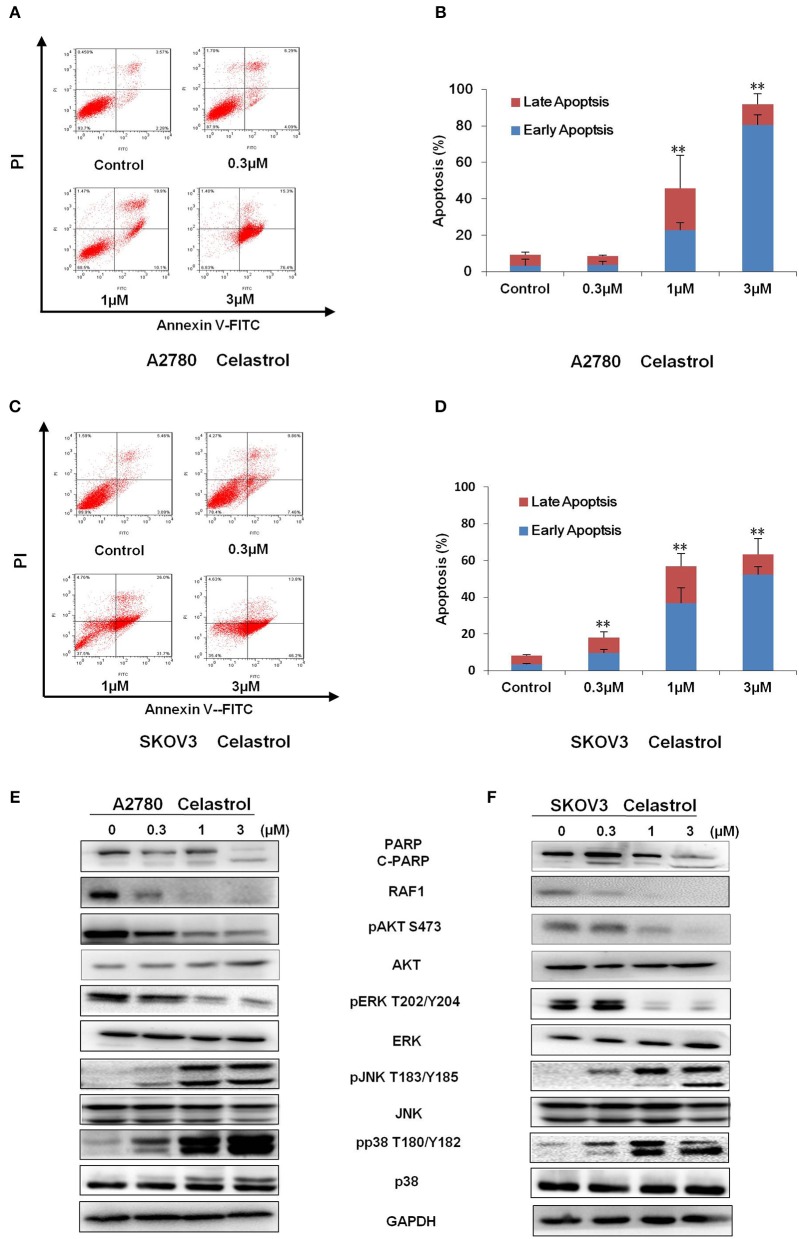
Celastrol induced apoptosis in ovarian cancer cells. A2780 and SKOV3 cells were treated with celastrol with the indicated concentrations for 48 h, then cell apoptosis was detected by FCM. The representative charts **(A,C)**, quantified data **(B,D)**, and Western blot results **(E,F)** of three independent experiments are shown. The same GAPDH image of Figure 2 has been used as loading control. ^**^P < 0.01 vs. corresponding control.

To determine whether celastrol could induce cell apoptosis, A2780 and SKOV3 cells were treated with indicated concentrations of celastrol for 48 h, apoptosis was assessed by FCM with Annexin V/PI staining. As shown in Figures 3A–D, celastrol dose-dependently induced early stage of apoptosis (Annexin V+/PI–) and late stage of apoptosis (Annexin V+/PI+) in both cells. Treatment of celastrol upregulated the protein expressions of cleaved-PARP, pp38 T180/Y182 and pJNK T183/Y185 but downregulated the protein expressions of pERK T202/Y204, pAKT S473 and RAF1 (Figures 3E,F). Consequently, these results suggest that celastrol induces cell apoptosis in ovarian cancer cells.

### ROS Generation Was Critical for Celastrol-Induced Apoptosis in Ovarian Cancer Cells

**Figure 4:**
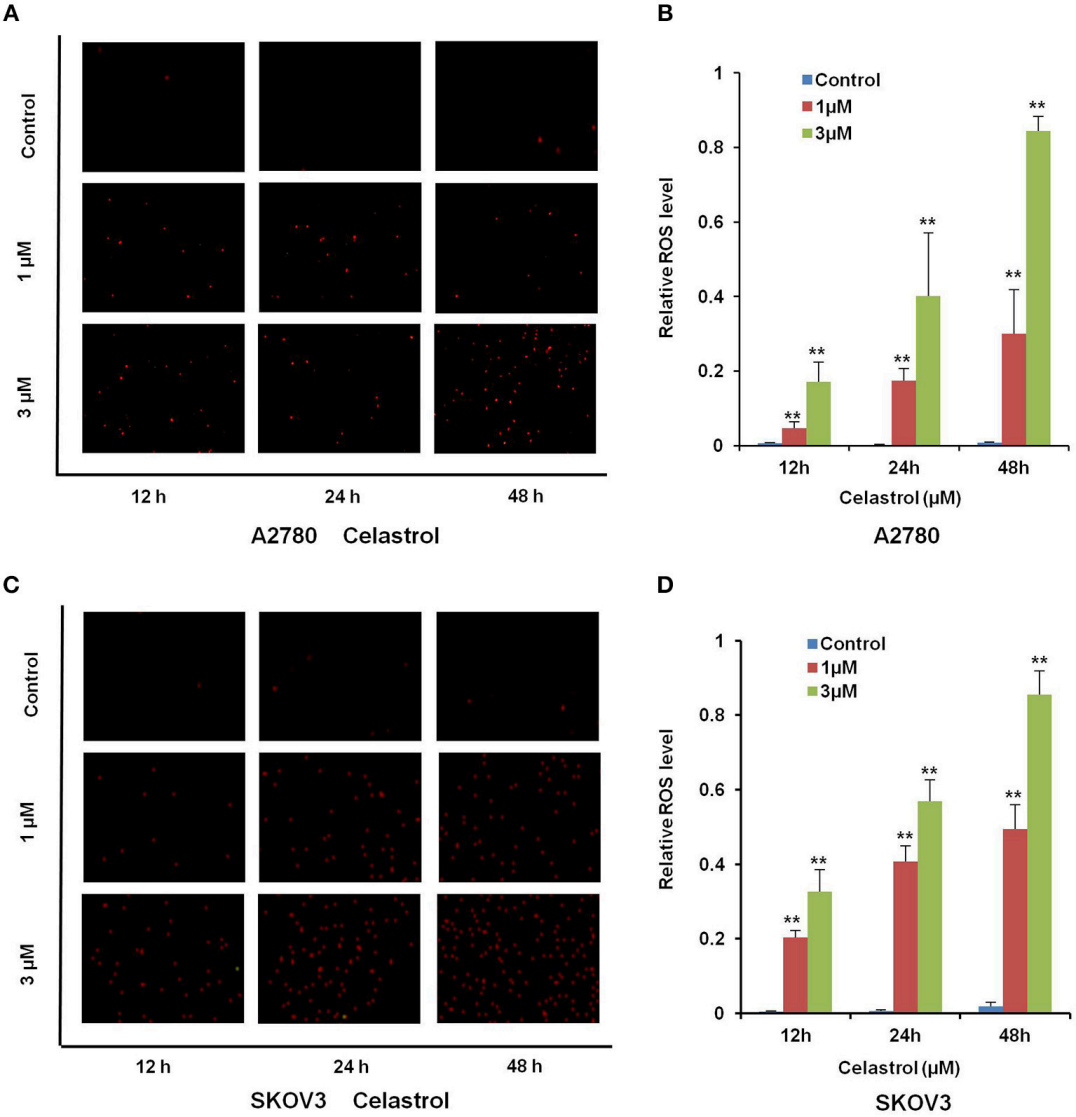
Celastrol enhanced the intracellular ROS levels in ovarian cancer cells. A2780 and SKOV3 cells were treated with celastrol with indicated times and concentrations, stained with DHE, photographed and quantified respectively under fluorescent microscope and FCM. The representative micrographs **(A,C)** and quantified results **(B,D)** were shown. ^**^P < 0.01 vs. corresponding control.

**Figure 5:**
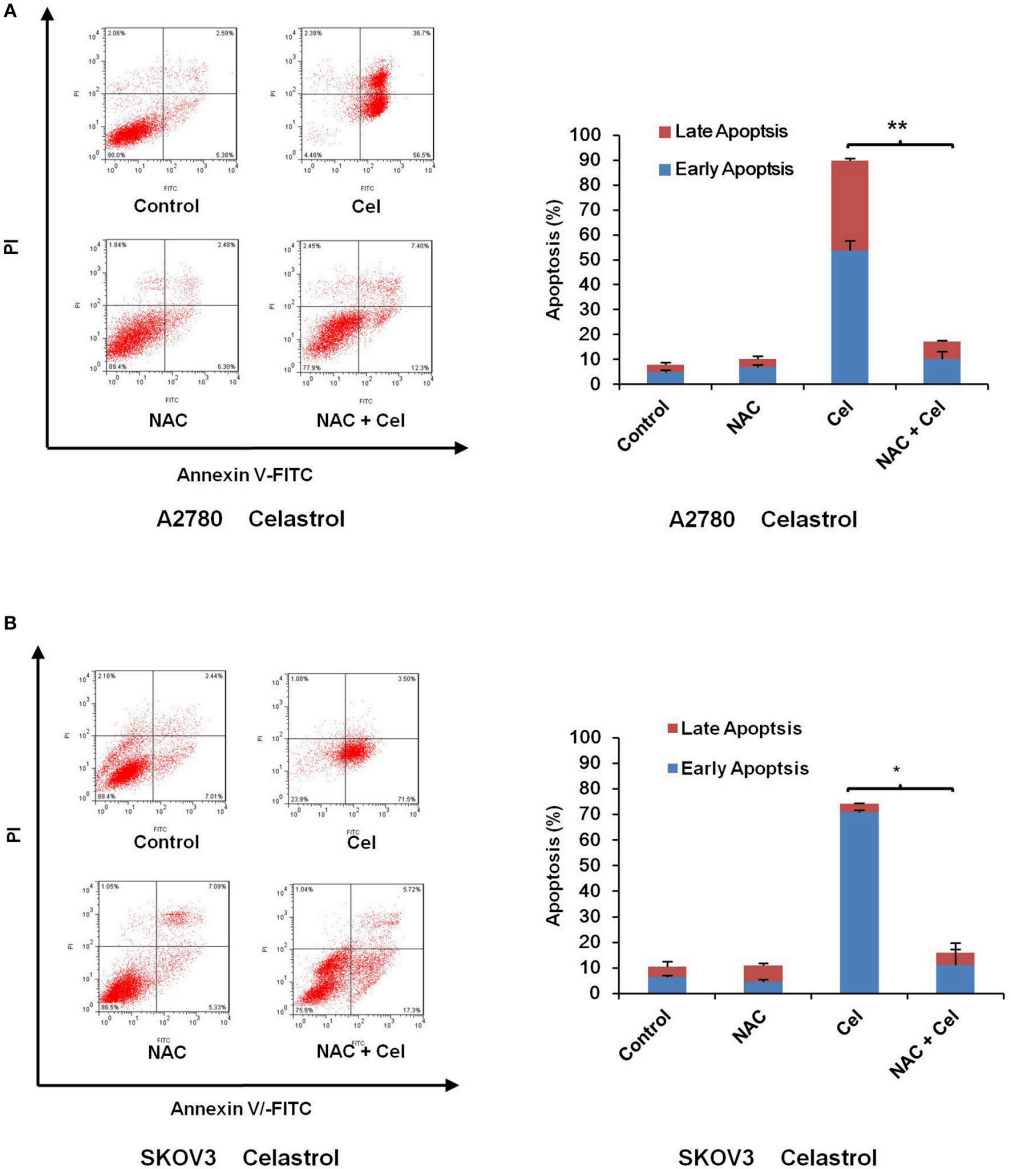
NAC impeded celastrol-induced cell apoptosis. A2780 and SKOV3 cells were treated with 3 μM celastrol for 48 h in the presence or absence of 5 mM NAC pretreated for 1 h. The apoptosis was detected by FCM. The apoptosis charts and quantified data **(A,B)** were shown. ^*^P < 0.05 and ^**^P < 0.01 vs. corresponding control.

Numerous antitumor agents demonstrate antitumor activity via ROS-dependent activation of apoptotic cell death (26, 27). It has previously been reported that the elevated intracellular ROS mediated celastrol-induced apoptosis in several human cancer cells (28). Thus, we surmised that celastrol caused apoptosis in ovarian cancer cells was due to excessive ROS generation. Firstly, the cellular ROS was tagged by DHE fluorescence staining in celastrol-treated cells. As shown in Figure 4, celastrol enhanced the detectable red fluorescent signals of DHE in both A2780 and SKOV3 cells, suggesting the intracellular ROS levels were increased after celastrol treatment. Then we pre-treated A2780 and SKOV3 cells with NAC (a specific ROS scavenger), Celastrol-induced cell apoptosis were totally attenuated by NAC in both ovarian cancer cells (Figure 5). Collectively, these results suggest that ROS generation was critical for celastrol-induced apoptosis in ovarian cancer cells.

### Celastrol Inhibited the Tumor Growth of Ovarian Cancer in Nude Mice

**Figure 6:**
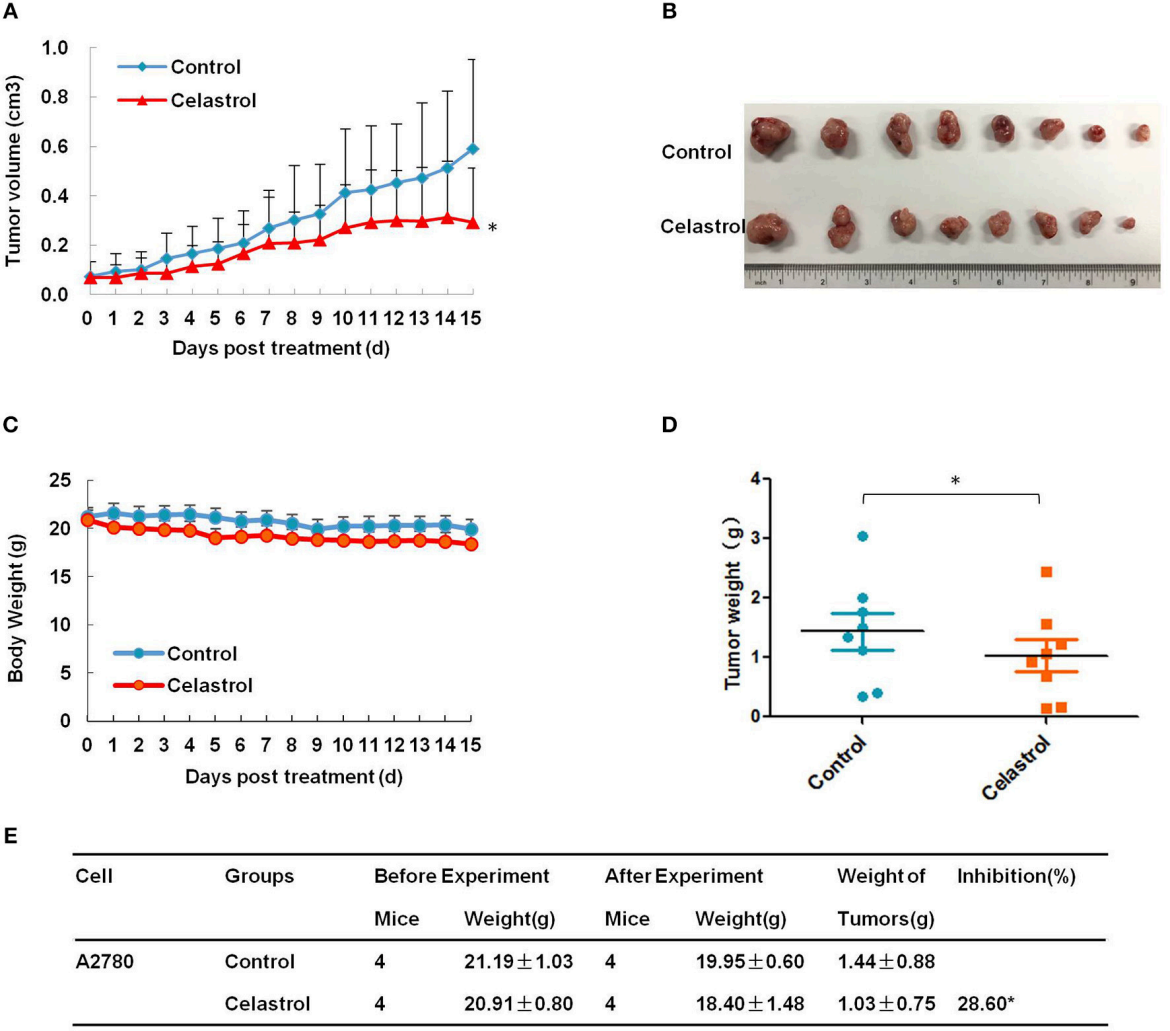
Celastrol inhibited the tumor growth of ovarian cancer in nude mice. Each mouse was injected subcutaneously with A2780 cells (4 × 10^6^ in 100 μl of medium) under the left and right shoulders. When the subcutaneous tumors were approximately 0.3 × 0.3 cm in size, mice were randomized into two groups, and received intraperitoneal injection of vehicle alone (0.5% methylcellulose) or celastrol (2 mg/kg) every day. The body weight and tumor volume were recorded every day. After experiment, the mice were anesthetized, and tumor tissue was excised from the mice and weighted. The tumor volume **(A)**, original tumors **(B)**, body weight **(C)**, tumor weight **(D)**, and summary data **(E)** were shown. ^*^P < 0.05 vs. corresponding control.

To confirm the antitumor effects of celastrol *in vivo*, A2780 subcutaneous xenograft tumors were generated in the nude mice. As shown in Figures 6A–E, treatment of celastrol did inhibit the growth of A2780 xenograft tumors with the inhibition ratio of 28.60% by diminishing the tumor volumes and weights. Furthermore, mice body weight in celastrol group was close to that of control group, suggesting that celastrol at the indicated dose did not cause toxicity in mice (Figure 6C).

## Discussion

Natural products attract more and more attention in the prevention and treatment of cancer in recent years. Products from the plant *Tripterygium wilfordii*, including celastrol and triptolide, are of special attention because of its superior anti-tumor activities against a variety of cancer types, and therefore are the traditional herb medicines considered to have the most potential in modern cancer therapy. For the treatment of ovarian cancer, triptolide has been shown to inhibit the proliferation, migration and invasion of ovarian cancer in multiple pathways (29–31) and demonstrated to exert efficacy in preclinical models (32). Celastrol has also been reported to induce apoptosis and inhibit proliferation, migration and invasion in ovarian cancer cells *in vitro* (14, 16), but the mechanism for its anti-tumor effect and the effect of celastrol on the growth of ovarian cancer cells *in vivo* are not fully understood. In our present study, we have demonstrated that celastrol mediated dose-dependent anti-growth effects on human ovarian cancer cell lines SKOV3 and A2780. The IC_50_ value after 72 h treatment with celastrol ranged from 2 to 3 μM in these two human ovarian cancer cell lines, similarly to the IC_50_ value of celastrol of ovarian cancer in other articles (15, 16). We have also shown that celastrol induced both the early and late stage of apoptosis and cell cycle arrest in G2/M phase with obvious up-regulation of cleaved-PARP, pp38 T180/Y182, pJNK T183/Y185, p27 and Cyclin B1 and down-regulation of pERK T202/Y204, pAKT S473, RAF1 and Cyclin E in a dose-dependent manner. Similar with our results, celastrol can induce the activation of JNK and inactivation of AKT in multiple myeloma cells RPMI-8226 (33), activation of p38 in ovarian cancer cells OVCAR-8 and colorectal cancer cells SW620 cells (34) and inactivation of ERK in hepatoma cells Hep3B (35). Furthermore, celastrol inhibited the growth of A2780 ovarian cancer subcutaneous xenograft tumors in nude mice by diminishing the tumor volumes and weights, and mice body weight in celastrol group was close to that of control group. These *in vitro* and *in vivo* data strongly indicate that celastrol may be a appropriate candidate for treating ovarian cancer.

Biological roles of ROS were intricate and important in cancer cells (36). The intracellular ROS plays a significant role in regulating multifarious cell physiological process such as growth, differentiation, death and so on (37). ROS changes the cellular redox condition, induces DNA damage and influences the activities of tumor suppressor or oncogene, thereby involving in the initiation and progression of cancer (38, 39). Lots of studies have shown that cancer cells normally produce more ROS than normal cells (40). Interestingly, accumulating evidence suggests that cancer cells are more vulnerable to ROS-induced death because they are under the increased oxidative stress (41). A variety of agents like YM155, dinaciclib and triptolide may be selectively toxic to tumor cells because they enhanced intracellular oxidant stress and push these already stressed cells beyond their limitation (24, 38, 42, 43). In addition, previous studies have demonstrated that ROS plays a pivotal role in celastrol-induced apoptosis in multiple cancers, such as colon cancer, liver cancer, osteosarcoma, etc. (9, 28, 44). In this study, we have found that the intracellular ROS levels were increased after celastrol treatment, and pre-treated with ROS scavenger NAC totally attenuated celastrol-induced cell apoptosis in ovarian cancer cells. It has been reported that celastrol enhanced the intracellular ROS to induce apoptosis by inhibiting mitochondrial respiratory chain complex I activity in lung cancer H1299 cells (45). Whether celastrol induces ROS accumulation to trigger apoptosis in the same way in ovarian cancer cells need to be further investigated.

In summary, our data have shown that celastrol induced cell growth inhibition, cell cycle arrest in G2/M phase and apoptosis with the increased intracellular ROS accumulation in ovarian cancer cells *in vitro* and *in vivo*. Pretreatment with NAC totally blocked the apoptosis induced by celastrol. Altogether, these findings suggest celastrol is a potential therapeutic agent for treating ovarian cancer.

## Author Contributions

L-NX, NZ, J-YC, ZS, and X-JY designed the experiments, performed the experiments, analyzed the data, and wrote the paper. X-WN, H-HZ, P-PY, Q-WJ, YY, J-RH, M-LY, Z-HX, M-NW, and YL performed the experiments. All authors read and approved the final manuscript.

### Conflict of Interest Statement

The authors declare that the research was conducted in the absence of any commercial or financial relationships that could be construed as a potential conflict of interest.
